# Relationship between Changes in Microbiota and Liver Steatosis Induced by High-Fat Feeding—A Review of Rodent Models

**DOI:** 10.3390/nu11092156

**Published:** 2019-09-09

**Authors:** Saioa Gómez-Zorita, Leixuri Aguirre, Iñaki Milton-Laskibar, Alfredo Fernández-Quintela, Jenifer Trepiana, Naroa Kajarabille, Andrea Mosqueda-Solís, Marcela González, María P. Portillo

**Affiliations:** 1 Nutrition and Obesity Group, Department of Nutrition and Food Science, University of the Basque Country (UPV/EHU) and Lucio Lascaray Research Institute, 01006 Vitoria, Spain; 2 CIBEROBN Physiopathology of Obesity and Nutrition, Institute of Health Carlos III, 01006 Vitoria, Spain; 3 Nutrition and Food Science Department, Faculty of Biochemistry and Biological Sciences, National University of Litoral and National Scientific and Technical Research Council (CONICET), Santa Fe 3000, Argentina

**Keywords:** dietary fat, steatosis, gut microbiota, dysbiosis, rodent, liver

## Abstract

Several studies have observed that gut microbiota can play a critical role in nonalcoholic fatty liver disease (NAFLD) and nonalcoholic steatohepatitis (NASH) development. The gut microbiota is influenced by different environmental factors, which include diet. The aim of the present review is to summarize the information provided in the literature concerning the impact of changes in gut microbiota on the effects which dietary fat has on liver steatosis in rodent models. Most studies in which high-fat feeding has induced steatosis have reported reduced microbiota diversity, regardless of the percentage of energy provided by fat. At the phylum level, an increase in *Firmicutes* and a reduction in *Bacteroidetes* is commonly found, although widely diverging results have been described at class, order, family, and genus levels, likely due to differences in experimental design. Unfortunately, this fact makes it difficult to reach clear conclusions concerning the specific microbiota patterns associated with this feeding pattern. With regard to the relationship between high-fat feeding-induced changes in liver and microbiota composition, although several mechanisms such as alteration of gut integrity and increased permeability, inflammation, and metabolite production have been proposed, more scientific evidence is needed to address this issue and thus further studies are needed.

## 1. Introduction

Nonalcoholic fatty liver disease (NAFLD) is the most common liver disease worldwide, affecting 20%–30% of the general population [1]. It is characterized by intra-hepatocyte triglyceride (TG) in more than 5% of hepatocytes, in the absence of significant alcohol consumption [2]. NAFLD includes a spectrum of chronic liver diseases starting with isolated simple steatosis, progressing to nonalcoholic steatohepatitis (NASH), fibrosis, and cirrhosis, which can lead to hepatocellular carcinoma (HCC) [3]. The histological techniques commonly used to characterize NAFLD progression are hematoxylin-eosin staining, Oil Red O-staining and Brunt’s classification [4]. Therefore, Brunt’s classification is a system for grading NAFLD depending on the histological lesions in mild (grade 1: fat occupying less than 30% of hepatocytes), moderate (grade 2: excess of fat affects between 30% and 60% of hepatocytes) and severe steatosis (grade 3: more than 60% of hepatocytes affected).

Liver steatosis results from increased flux of free fatty acids (FFAs) to the liver, due to obesity and insulin resistance, and/or increased *de novo* lipogenesis. In the presence of excessive amounts of FFAs, increased levels of ß-oxidation leads to enhanced production of reactive oxygen species (ROS), and thus oxidative stress [5]. As a consequence, lipid peroxidation by-products are produced [6] and this leads to the synthesis of pro-inflammatory cytokines, promotes influx of inflammatory cells into the liver, and activates stellate cells, leading to collagen deposition [5]. These events explain the progression of liver steatosis to NASH).

A great number of studies have observed that gut microbiota can play a crucial role in NAFLD and NASH development, both in rodents and humans. Although more than 1000 bacterial species in the intestine have been described so far, two phyla, *Firmicutes* and *Bacteroidetes,* predominate, making up 90% of the total [7]. However, not only these phyla play an important role in NAFLD progression, also other bacteria, such as *Proteobacteria, Verrucomicrobia, Actinobacteria, Fusobacteria,* and *Cyanobacteria* may be relevant [8,9]. Regarding patients suffering NAFLD, different patterns of gut microbiota have been described in those with simple steatosis. Moreover, differences have also been observed between patients with simple steatosis or steatohepatitis [10,11,12] and between those with or without fibrosis [12].

It has been suggested that alterations in gut microbiota composition in NAFLD and NASH may lead to the impairment of hepatic TG metabolism and be linked to the hepatic inflammatory state associated with this condition [13,14]. Nevertheless, due to the great differences among the results reported in clinical studies, in all likelihood due to differences in experimental designs, it cannot be concluded which differences in microbiota composition may be expected [15]. For example, while decreased *Bacteroidetes* have been described in NASH in some studies [10], increases in this parameter have been reported in others [16].

The gut microbiome is influenced by different environmental factors, among others diet and lifestyle. As far as dietary fat is concerned, both its amount and its quality can modify microbiota composition. Under physiological conditions, small intestine offers a physical and biological barrier that prevents bacteria and toxin from getting through. This barrier is assisted by the tight-junction proteins, such as occludin, zonula occludens-1 (ZO-1) and claudin 2, and controls the permeability of the paracellular transport pathway. High-fat (HF) diets can disturb the integrity of gut epithelium by reducing the amount of these proteins. This leads to the increase on gut permeability and then to endotoxemia and systemic inflammation [17]. Furthermore, it seems that diets with a high content in saturated, monounsaturated or polyunsaturated fats may also have different effects on microbiota [18].

The aim of the present review is to summarize the information provided in the literature concerning the impact of changes in gut microbiota on the effects of dietary fat on liver steatosis in rodent models. For this purpose, first a summary of each study, grouping them according to dietary fat source is presented. Afterwards, the mechanisms underlying the relationship between microbiota modification and diet mediated liver composition changes are explained.

## 2. Studies Carried Out by Using Lard as the Main Dietary Fat Source

Most of the studies consulted for this review used lard as a dietary fat source (Table 1). Some of them have been carried out in mice and others in rats. Using mice as animal model, and beginning with those in which very HF diets were used (60% of energy from fat), Gauffin Cano et al. [19] fed male C57BL-6 mice a HF diet for 7 weeks. The authors observed a greater hepatic TG content in the HF diet-fed animals when compared to the control group, which was confirmed histologically, according to Brunt’s classification [4].

As far as fecal microbiota is concerned, HF diet feeding induced reductions in *Lactobacillus* and *Bifidobacterium*, as well as in *Clostridium coccoides* and *Clostridium leptum* while increased the *Enterobacteriaceae* family. These changes led to the enhancement of pro-inflammatory signals coming from the gut, as observed by the higher production of tumor necrosis factor α (TNF-α), which could affect the liver resulting in NAFLD [20]. When *B. uniformis* CECT 7771 was administered to the animals used in this study, both HF diet-induced intestinal dysbiosis and hepatic steatosis ameliorated.

More recently, Wang et al. [21] carried out a study in Institute of Cancer Research (ICR) mice fed either a normal-fat diet (NF group, 10% of calories from fat) or a HF diet (HF group, 60% of calories from fat, mainly lard) for a shorter period (4 weeks). The liver lesion score reported for the HF group (2.3 ± 0.3) was significantly higher than that for the normal diet (ND) group (which was 0). Moreover, the greater liver damage reported in the HF group was also reflected by a greater gene expression of interleukin-1β (*Il-1β*), a widely used marker of inflammation.

Microbiota analysis demonstrated that HF diet feeding significantly altered its composition, by increasing *Firmicutes* and decreasing *Bacteroidetes* population, thus resulting in a significantly lower *Bacteroidetes/Firmicutes* ratio. Moreover, significantly augmented *Deferribacteres* population was also observed in the HF diet group, in comparison to the NF group. At the class level, the main changes decreased *Bacteroidia* and increased *Clostridia* and *Deferribacteres* populations in the HF diet-fed group. Finally, regarding the family level, HF diet feeding significantly increased the populations of *Ruminococcaceae, Lachnospiraceae*, and *Bacteroidaceae,* while lowering *Bacteroidales S24-7*. These results demonstrate that HF feeding dysregulates gut microbiota composition. Based on these results, the authors suggested that the greater liver lesion induced by a HF diet could be due to changes in gut microbiota composition along with greater gene expression of inflammatory markers.

In another study, Xu et al. [22] fed male C57BL/6J mice with a normal-fat diet (NC group) or a HF diet (HF group, 60% of energy from fat) for 10 weeks. Both the histopathological analysis and liver TG quantification demonstrated higher hepatic lipid accumulation in the HF group than in the NC group. Similarly, greater protein expressions of toll-like receptor 4 (TLR4) and nuclear factor kappa B (NF-κB), both involved in signaling pathways associated with inflammation, were found in the HF group in comparison to NC group.

Regarding the microbiota, data showed that although no significant differences were found in gut microbiota richness between both experimental groups, in the HF group the *Firmicutes/Bacteroidetes* ratio was higher. Moreover, greater relative abundance of harmful microbes, such as *Helicobacter marmotae*, *Odoribacter, and Anaerotruncus,* was also appreciated in this group. Finally, when the gastrointestinal track was studied, changes in its morphology (shorter small intestine length, and lower small intestine/body weight and colon/body weight ratio values) were found as a result of the HF feeding. Based on these results, the authors concluded that the HF feeding-derived dysbiosis could be related to hepatic steatosis development through the induction of a low-grade inflammation.

In the study reported by Su et al. [23] BALB/c male mice were fed a NF or a HF diet for a long period of time (18 weeks). Mice fed the HF diet showed severe liver steatosis compared to the control group. The authors also reported that the HF group presented greater liver CD3+ lymphocyte infiltration than did control mice. As hepatic steatosis can be a consequence of insulin resistance, homeostatic model assessment of insulin resistance (HOMA-IR) index was calculated. Therefore, HF feeding led to higher HOMA-IR.

To know whether HF feeding could promote endotoxemia, which could lead to liver inflammation and hepatic steatosis, the authors measured plasma lipopolysaccharide (LPS) and found a higher level in HF group. Moreover, tight-junction proteins occludin, (ZO-1) and claudin 2, critical for the maintenance of the intestinal epithelial barrier, were significantly lower in HF group than in control mice. As far as microbiota composition is concerned, *Firmicutes* bacteria augmented, whereas *Bacteroidetes* were reduced by the HF diet, thus resulting in a significantly lower *Bacteroidetes/Firmicutes* ratio. However, *Proteobacteria* slightly increased in control mice. Furthermore, it was found that *Helicobacter hepaticus*, which has been reported to induce liver tumors and hepatitis in certain immunodeficient mice [48], was sharply elevated (6000-fold) in the lumen of HF mice. By contrast, the *Akkermansia muciniphila* bacteria, which belongs to the *Verrucomicrobia* phylum and are inversely correlated with diabetes and the metabolic syndrome, decreased in the intestinal microbiota of mice receiving the HF diets.

Recently, Porras et al. [35] carried out a study in male C57BL/6J mice fed a NF diet (10% energy from fat) or a HF diet (HFD, 60% energy from fat, mainly lard) for 16 weeks. When the hepatic function was evaluated, elevated hepatic TG and free fatty acid (FFA) levels were found in mice fed the HF diet. Impaired lipid metabolism was confirmed with a 10-fold increase in NAFLD activity score (NAS), greater inflammation and ballooning in rats being fed in the HF diet. The development of hepatic steatosis was accompanied by the rise of lipid peroxidation and higher cytochrome P450 2E1 (*Cyp2e1*) gene expression in HF mice compared with the control group. Along the same line, a relationship between CYP2E1 and endoplasmic reticulum (ER) stress, contributing to NAFLD progression, was stablished.

Microbiota analysis revealed that *Firmicutes* and *Proteobacteria* expansion was higher in the HF mice than in the control group. By contrast, *Bacteroidetes* phylum was significantly reduced when compared to the control mice, thus increasing the *Firmicutes/Bacteroidetes* ratio. At the class level, it was found that *Clostridia* and *Bacilli* (both belonging to the *Firmicutes* phylum) and *Deltaproteobacteria* grew with the HF diet, whereas *Bacteroidia* (*Bacteroidetes* phylum), *Erysipelotrichi* (*Firmicultes* phylum) and *Betaproteobacteria* (*Proteobacteria* Phylum) showed a diminished expansion compared with the control mice. Finally, more than 400 genera of known bacteria were detected in gut microbiota. Thus, HF mice presented a higher colonization of *Desulfovibrio, Blautia, Oscillospira*, and *Lactobacillus* bacteria. A sharp increase in *Helicobacter* expansion was also observed in the HF diet-fed animals. By contrast, some bacteria genera such as *Parabacteroides* and *Alkaliphilus* showed reduced levels in HF diet-fed mice. The authors suggested that liver steatosis was positively correlated with *Firmicutes/Bacteroidetes* ratio, although negatively with total bacteria concentration.

To gain more insight into the relationship between steatosis and HF feeding produced changes in microbiota, the authors measured short-chain fatty acids (SCFAs), which can improve gut barrier integrity. In this regard, a reduction in acetate (−32%), butyrate (−29%) and propionate (−21%) SCFAs was observed with the HF diet. These results are in accordance with the diminution of the claudin-1 and occludin intestinal tight-junction proteins, as well as with the reduction of intestinal alkaline phosphatase alkaline. Indeed, alkaline phosphatase is related to the detoxification of bacterial LPS, controlling the gut inflammation. Thus, LPS levels, related to dysbiosis, increased (+73%), as well as ethanol levels (+34%) in plasma of mice consuming the HF feeding. In addition, up-regulation of hepatic *Tlr-4,* interleukin-6 (*Il-6*) and *Tnf-α* gene expressions, as well as activation of both the NF-κB pathway and the hepatic NLRP3 inflammasome component, were observed in these animals. This was due to the fact that NAFLD development could be regulated by the inflammasome response, which is associated with both the TLR-NF-κB pathway induced dysbiosis and the oxidative stress-related lipotoxicity.

In the study carried out by Jing et al. [38] in male C57BL/6J mice, animals were assigned to a normal-fat-fed group (NC; 10% of energy from fat) or a HF-fed group (60% of energy from fat). After 11 weeks of dietary intervention, histological analysis showed that HF-fed mice had greater lipid deposition and microvesicular steatosis in the liver, with small lipid droplets in cells. Gut microbiota composition differed significantly between both experimental groups. In general terms, species diversity and richness decreased in HF-fed mice. At the phylum level, the HF group showed a marked decrease in the relative abundance of *Firmicutes* (3.63 versus 19.32%) and an increase in the relative abundance of *Verrucomicrobia* (31.61 versus 0.01%) when compared to the NC group.

More recently, Wu et al. [47] carried out an experiment in C57BL/c mice fed either with a NF diet or a HF diet (60% of energy from fat) for 12 weeks. The excessive saturated fat consumption significantly increased TG and cholesterol content in the livers of the HF diet-fed animals. Moreover, histological analysis revealed ballooning. In contrasts to reports by other studies, when gut microbiota was analyzed, no differences were observed in the diversity, as estimated by Shannon, Chao1, and Ace indexes. Principal Coordinate Analysis (PCoA) and the average clustering of the microbial communities revealed differences between the two experimental groups, suggesting that the HF diet modified the structure of the microbial community. The microbiota analysis revealed a decrease in the phylum *Bacteroidetes* phylum without changes in *Firmicutes* in HF diet-fed animals. At the genus level, an increase in *Anaerotruncus* and *Streptococcus*, and a decrease in *Bacteroides* were observed in this group.

Other studies have used rats as animal model instead of mice. In this regard, Liu et al. [28] aimed to study the effects of different diets, HF or high-sugar and high-protein diets, on intestinal microbiota expansion and NAFLD development. For this purpose, male Sprague–Dawley rats were fed a control diet (16.5% lard), a HF diet (60% lard) or a high-protein diet (60% casein and 16.5% lard) for 12 weeks. Focusing on HF diet-induced effects, the HF-fed group showed higher liver weight than the control animals did at the end of the experimental period. Similarly, serum transaminase levels and liver TG content were also higher in this group. Histological analysis confirmed this fact.

As far as microbiota analysis is concerned, *Firmicutes, Bacteroidetes*, *Proteobacteria, and Tenericutes* were the most abundant of a total of 15 phyla. Thus, rats fed HF diet presented higher *Firmicutes* expansion and lower amount of *Bacteroidetes* bacteria (lower *Bacteroidetes* to *Firmicutes* ratio). Regarding the genus level, rats receiving HF feeding showed greater *Roseburia* and *Oscillospira* expansion, whereas *Bacteroides* and *Parabacterioides* genera decreased. Finally, LPS levels that were analyzed in portal vein blood were significantly higher in rats consuming HF feeding. Thus, LPS level was considered a triggering factor in the development of metabolic disorders.

In another study carried out in rats, Chen et al. [36] fed male Sprague–Dawley rats with either in a standard diet (10% of energy from fat) or a HF diet (60% energy from fat) for 12 weeks. In the standard diet, the fat sources were soybean and lard (in similar proportions), while in the case of the HF diet the fat was mainly lard (although a small quantity of soybean fat was also present in order to provide essential fatty acids). At the end of the experimental period, the rats fed the HF diet showed significantly higher body weight and serum levels of alanine aminotransferase (ALT) and aspartate aminotransferase (AST), when compared to the control group. Greater fat droplet accumulation and ballooning were found in hepatocytes from the HF diet-fed group. As far as liver TG content is concerned, this parameter was elevated in the HF group. Moreover, regarding oxidative stress, a process involved in the progression of hepatic steatosis to more harmful stages, no differences were found in malonaldehyde (MDA) levels. By contrast, significantly lower activity of the antioxidant enzyme superoxide dismutase (SOD) was found in the HF group.

When microbiota composition was determined, at the phylum level, increased relative abundance of *Proteobacteria* and *Verrucomicrobia* was found in the HF group when compared with the control group. By contrast, the relative abundances of *Bacterioidetes* and *Tenericutes* were lower. At the genus level, 45% of the total operational taxonomic units was identical in control and HF groups. The results obtained show that a HF feeding pattern could shift microbiota composition towards a less diverse and abundant one. Indeed, the authors point towards the relationship between the gut bacteria in the host and the development of health alterations such as NAFLD, obesity, and inflammation.

In addition to these animal models, in several studies knock out (KO) mice have been used to analyst the role of specific genes. Thus, Duparc et al. [33] assessed the role of hepatocyte myeloid differentiation primary response gene 88 (MyD88), which is linked to several metabolic pathways, on intestinal microbiota composition and hepatic function. For this purpose, wild-type (WT) and hepatocyte-specific Myd88 deleted (LKO) mice (generated from C57BL/6 mice) were fed a NF diet (10% energy from fat) (WT-CT or LKO-CT groups) or a HF diet (HF, 60% energy from fat, mainly lard) (WT-HF or LKO-HF groups) for 8 weeks. At the end of the experimental period, greater liver weight and hepatic TG content were observed in the WT mice fed the HF diet. Regarding the hepatic function, plasma ALT level was drastically increased in mice fed the HF diet. As a result, the steatosis score was significantly higher in the WT-HF group than in the control group, which was also confirmed by histopathological analysis.

Since HF feeding is related to impaired intestinal barrier functions, several markers of gut microbiota were assessed in the jejunum and ileum. Thus, no differences were found in the tight-junction proteins (claudin 1 and 3, ZO-1, and occludin). The bacterial richness was similar in both experimental groups, but in contrast to most of the reported studies, diversity was greater with HF feeding. Regarding the bacteria phyla detected in the microbiota, *Firmicutes* significantly augmented with the HF diet, whereas *Tenericutes* decreased. However, no differences were observed in *Bacteroidetes* phylum between WT-CT and HF-CT groups. Concerning the bacteria genera, *Sutterella* and *Allobaculum* expansion were reduced in rats fed in the HF diet, whereas *Ruminococcus* and *Oscillospira* augmented.

Regarding the hepatocyte-specific deletion of MyD88, a decrease in insulin resistance, a lower steatosis score and a reduction in *Firmicutes* levels were appreciated in LKO-CT group compared to WT-HFD group. Moreover, the MyD88 deletion worsened the glycemic control induced by the HF diet. The authors suggested that the genetic deletion of MyD88 in hepatocytes can modify intestinal microbiota composition and hepatic functions.

Martins et al. [41] addressed the role of interleukin-23 (IL-23) in driving the intestinal T helper type 17 response during obesity and metabolic syndrome progression induced by a HF diet. For this purpose, female C57BL/6 mice deficient in IL-23 and their littermate controls were used. The animals were divided into the following groups: (1) group I, wild-type (WT) mice fed a NF diet, (2) Group II, IL-23-deficient mice fed the HF diet, (3) group III, WT mice fed a HF diet, which provided 60% of energy from fat, mainly lard, and (4) group IV, IL-23-deficient mice fed the HF diet. C57BL/6 mice and IL-23-deficient mice were fed NF or HF diet for 20 weeks. Histological analysis showed that IL-23 deficient mice fed the HF diet exhibited a greater fat accumulation in the liver than the WT mice fed the HF diet.

Regarding microbiota, the WT mice fed the HF diet-fed showed an increase in *Lactobacillaeceae* and *S24-7* and a decrease in *Lachnospiraceae* compared to mice in WT/CTD group. Considering that gut dysbiosis is intimately related to bacterial translocation and gut permeability, they analyzed whether the dysbiosis induced by IL-23 deficiency had any effect on the gut epithelial barrier. As expected, the results showed higher gut permeability, measured by FITC-Dextran, in HFD-fed IL-23p19^−/−^ mice compared with WT mice and a trend to greater LPS levels. The researchers concluded that the inhibition of IL-23 had a crucial role in limiting gut microbiota dysbiosis and maintaining gut epithelial barrier integrity, resulting in lower bacterial translocation.

Jia et al. [37] published a study where male C57BL/6 J mice were fed either a NF diet or a HF diet (60% kcal from fat, mainly lard) for 28 weeks. Histologically, animals fed the HF diet group presented increased lipid droplet and ballooning injury, and consequently, greater steatosis and lobular inflammation. Hepatic TG and total cholesterol amounts, as well as transaminase levels, were also higher than those reported in the control group.

Mice fed the HF diet showed lower Shannon index, which means that there was lowered alpha bacterial diversity. PCoA analysis demonstrated a clear difference between both groups. At the phylum level, an increase in the relative abundance of *Firmicutes,* without changes in that of *Bacteroidetes* and *Proteobacteria* was observed. In the *Firmicutes*, the genera *Eubacterium, Blautia, Clostridium*, and *Lactobacillus* were augmented in HF group. Moreover, in *Proteobacteria* phylum, an increase in *Escherichia* and a decrease in *Parasutterella* genus were observed.

Wu et al. [43] fed C57BL/6 mice either a NF or a HF diet for 18 weeks. HF feeding led to an increase in hepatic TG levels. Histological analysis confirmed fatty liver development. When gut microbiota was analyzed the authors observed that PCoA showed a clear difference between control and HF group, without changes in Shannon index. At the class level, a higher abundance of *Deltaproteobacteria* and *Deferribacteres* was observed in mice fed the HF diet, without changes in *Actinobacteria*. In the case of the family level, HF-fed animals showed increased relative abundance of *Desulfovibrionaceae, Deferribacteraceae*, and *Porphyromonadaceae*. Finally, at the genus level, no changes were observed in the relative abundance of *Bifidobacterium.*

In a recent study, Chen et al. [44] aimed to illustrate the function of high mobility group box 1 (HMGB1) in the gut microbiota and NAFLD. For that purpose, they carried out an experiment in male or female C57BL/6J mice WT and apoptosis-associated speck-like protein containing a C-terminal caspase recruitment domain (ASC) knock-down (ASC-KO). Animals were distributed in 4 experimental groups: (1) ND WT: wild-type mice fed a normal diet, (2) ND ASC-KO: ASC-KO mice fed a normal diet, (3) HF WT: wild-type mice fed a HF diet and (4) HF ASC-KO: ASC-KO mice fed a HF diet. The HF diet provided 60% energy from fat (mainly lard). The experimental period length was of 8 weeks.

The effect of the HF diet on lipid infiltration was confirmed. To determine histological liver damage, NAS, which involves steatosis, lobular inflammation, and hepatocellular ballooning, was calculated. Higher values were observed in animals fed the HF diet when compared to the NF diet, as well as in ASC-KO mice rather than in WT mice. Regarding gut microbiota abundance, at the phylum level the bacterial diversity lessened in the HF ASC-KO group. As expected, the HF diet augmented *Firmicutes* and reduced *Bacteroidetes*, increasing the *Firmicutes/Bacteroidetes* ratio. These changes were more prominent in ASC-KO than in WT mice. HF ASC-KO mice showed more *Streptococcaceae* than ND ASC-KO mice. Taken together, these results indicate that HF ASC-KO rodents had greater gut dysbiosis than the other groups.

Gut histological modifications were also evaluated. When comparing the four groups, mice fed a HF diet showed greater values, the damage being again greater in the ACS-KO mice. To confirm whether intestinal mucosal damage was caused by dysbiosis, the authors carried out an immunohistochemical analysis of HMGB1 protein, a key mediator of intestinal inflammation in NAFLD. HMGB1 values were higher in HF-fed mice than in ND-fed ones, and in ASC-KO mice than in WT ones. Using a confocal microscope, they observed an extensive co-localization of HMGB1 and CD63, a classical marker of exosomes, in the intestine. Co-localization was greater in HF mice and even more in ASC-KO mice. In this line, they measured HMGB1 in serous exosomes and its levels were higher in animals fed HF diet and even more in HF ASC-KO mice. The authors concluded that hepatic steatosis might be exacerbated by the HMGB1 of the exosomes released from the gut. However, they also explained that a confirmation of the crosstalk action of HMGB1 between the gut and liver is required.

Pathogen-free animals were also used by Wang et al. [26] in a study in which male C57BL/6J mice were fed either a standard or a HF diet (60% energy as fat, with lard as the main source) for 10 weeks. HF-fed mice showed several hallmarks of the metabolic syndrome including hepatic steatosis, as confirmed by histomorphology. In addition, an altered microbiome was observed as compared with their chow diet-fed counterparts. Moreover, an increase in *Tnf-α* gene expression was observed in liver after HF feeding.

The changes observed in microbiota after HF feeding increased abundances of *Firmicutes* and *Proteobacteria* and diminished abundances of *Bacteroidetes* and *Actinobacteria*. Therefore, the *Firmicutes/Bacteroidetes* ratio was higher upon HF diet. To establish associations between microbiota composition and the hepatic steatosis as a hallmark of the metabolic syndrome, the authors performed a principal component analysis (PCA). They found that HFD-induced changes in microbiota were significantly correlated with liver *Tnf-α* mRNA levels. Moreover, HF feeding led to a disruption in acetate production by gut microbial fermentation. Acetate has been shown to suppress body fat accumulation and inflammation in obese rodents which increases LPS plasma concentrations and hepatic *Tnf-α* gene expression, both related to an increased hepatic fat accumulation.

Chiu et al. [32] carried out an interesting study devoted to analyzing the role of gut microbiota in NAFLD development, using germ free mice (GF) which were colonized with fresh feces from healthy individuals or patients with NASH. For this purpose, male GF C57BL/6JNarl mice were orally inoculated with fecal mixtures of healthy humans (HL) or NASH patients (NASH). Disease development was checked in the donors by measuring serum biochemistry parameters (transaminases, TG and fasting glucose) and abdominal ultrasonography, as well as hepatic biopsy (only in the NASH group). Having confirmed that the animals indeed developed the disease, each group (HL and NASH) were fed a commercial standard diet (10% of calories from fat, lard and soybean on similar amounts) and HF diet (60% of calories from fat, mainly lard) for 16 weeks, thus resulting in four experimental groups: (a) HL fed a standard diet (HL-ST), (b) HL fed a HF diet (HL-HF), (c) NASH fed a standard diet (NASH-ST) and (d) NASH fed a HF diet (NASH-HF).

Once the experimental period was completed, no differences were found in body weight or in liver weight between HL-HF and NASH-HF groups. This lack of changes in these parameters was also observed between the groups fed in the ST diet (HL-ST and NASH-ST). As far as serum transaminase concentrations are concerned, ALT and AST levels were significantly increased in the NASH-HF group when compared to the HL-HF group, while in the case of the ST diet-fed animals only greater levels of AST were also appreciated in NASH-ST group when compared to the HL-ST group, without differences in AST levels. In the same line, greater hepatic TG content was found in the NASH-HF group when compared to the HL-HF group. A similar pattern was also found in the ST diet-fed animals, liver TG content being significantly higher in the NASH-ST group. Moreover, histological analysis, carried out using hematoxylin and eosin and Oil Red O-staining, showed a more advanced stage of steatosis and multifocal necrosis, as well as a greater amount of stained lipid droplets, in the NASH-HF group than in the HL-HF group. Regarding ST diet-fed animals, mild steatosis accompanied by moderate necrosis was observed in the NASH-ST group. As expected, neither lipid droplets nor multilocular necrosis were found in the HL-ST group. Then, the authors analyzed the gene expression of several inflammatory markers in the livers. In this regard, greater gene expressions of toll-like receptor 2 (*Tlr2*), *Tnf-α* and monocyte chemoattractant protein 1 (*Mcp 1*) were found in the NASH-HF group in comparison to the HL-HF group. Regarding the ST fed animals, the only changes in gene expression were observed in *Il-6* and *Mcp 1* levels, which were significantly higher in the NASH-ST group than in the HL-ST group.

When gut microbiota composition was studied, the relative abundance of *Firmicutes* (*Lactobacillates, Streptococcaceae, Eubacteriaceae* and *Clostridiaceae*) was significantly higher in the NASH-ST group, as well as in the two groups fed in HF diet (HL-HF and NASH-HF) and the highest relative abundance of *Bacteroidetes* was found in the HL-ST group. Among the two HF diet-fed groups, at phylum level the relative abundance of *Proteobacteria* (*Enterobacteriaceae*) was significantly higher in the NASH-HF group; while at the family level, significant increases of *Streptococcaceae* relative abundance was also found in this group. Furthermore, up-regulation of *Enterobacteriaceae* was only observed in the NASH-HF group. In this regard, no differences in *Streptococcaceae* relative abundances were found between HL-ST and HL-HF groups. This points towards the potential association between the relative abundance of these microbes and the progression of NAFLD.

Based on the results obtained, the authors demonstrated that regardless of the diet used (ST or HF), the proportion of *Bacteroidetes* found in the gut microbiota of mice inoculated with feces of NASH patients decreased when compared with that of the mice inoculated with HL patient feces. Moreover, the reported results reported also suggest that the combination of NASH patient-derived microbiota with a HF diet feeding pattern could aggravate NAFLD progression. This observation was supported by the fact that the animals in the HL-HF group had only developed a simple steatosis, whereas in the NASH-HF group the severity of steatosis (macro and micro) was higher. The results also showed that mice inoculated with NASH feces had a greater proportion of *Firmicutes*. The authors concluded that the composition of intestinal microbiota may influence in the NAFLD or NASH progression, as demonstrated by the greater severity of NAFLD found in the gnotobiotic mice colonized by fecal bacteria of NASH patients. In this regard, the authors suggested that a complex cooperative effect among host gut microbiota, inflammatory cytokines, and TLRs could underlie these events.

Finally, specific pathogen-free (SPF) C57BL/6J male mice were also used in the study reported by Wang et al. [42]. Animals were randomly distributed into a control group fed a normal-fat diet and a group fed a HF diet for 14 weeks. Serum TG, cholesterol, and transaminases increased in HF diet animals, accompanied by a decrease in high-density lipoprotein (HDL) cholesterol levels. Liver weight was greater in mice from HF group and histological analysis revealed more lipid droplets in this group. Moreover, genes related to inflammation such as *Il-6* and *Mcp1* were up-regulated in this group of mice.

As far as microbiota is concerned, the authors observed an increase in the abundance of Gram-negative bacteria in animals fed HF diet, with no changes in the abundance of Gram-positive bacteria. More specifically, *Clostridium leptum* increased, whereas *Bacteriodetes* were lessened, without changes in *Escherichia coli*. Finally, an increase in endotoxin level was also observed in HF group.

In other studies fat represented 45% of total energy. Seo et al. [25] fed male C57BL/6 mice either a standard diet or a commercial HF diet (45% of energy as fat from lard) for 8 weeks. HF feeding elevated fat accumulation in the liver. Indeed, in the histological analysis, large vacuoles containing TG were evident in HF diet-fed animals as compared with those from standard diet.

Interestingly, the authors reported that HF diet induced an increase in the *Bacteroides/Prevotella* and *Firmicutes/Bacteroidetes* ratios, which have been pointed out as signature biomarkers for obesity and type 2 diabetes. Increased levels of *Firmicutes* in the gut allow the host to extract more energy from food, thus contributing to hepatic steatosis development.

Tung et al. [31] addressed a study in male C57BL/6 mice fed either a normal diet (15% energy from fat) or a HF diet (45% energy from lard-based fat) for 18 weeks. The livers of mice in the HF group presented a yellowish color and a higher weight, suggesting hepatic fat accumulation. Gut microbiota was modified by HF feeding. Thus, *Firmicutes* decreased by 33% in mice fed the HF diet compared with the control group, while *Bacteroidetes* doubled in quantity. Consequently, *Bacteroidales* and *Sphingobacteriales* population, belonging to *Bacteroidetes* phylum, tended to increase in HF diet-fed rats. Regarding the gut bacteria order, *Clostridiales* was the predominant bacteria in all the rat groups, though HF diet induced a 41% decrease in expansion.

At the same time, Leal-Díaz et al. [27], used male C57BL/6 fed a normal diet (control), or a HF diet containing 45% of energy from fat (7% *w*/*w* soybean oil and 16% *w*/*w* lard) for 12 weeks. No differences were detected in liver weight between control and HF rats. The rise in hepatic TG content confirmed that HF feeding-induced hepatic steatosis. To gain more insight into the effects on liver, an analysis of hepatic inflammation and oxidative stress showed higher TNF-α levels and macrophage infiltration under HF feeding conditions. Similarly, MDA content and ALT values were also greater in this group.

The Shannon index was higher in the control group. At the phylum level study, the results showed that changes in *Firmicutes* and *Bacteroidetes* were quite moderate. *Prevotella* genus, belonging to *Bacteroidetes* phyla, and *Mucispirillum* genus, (belonging to *Deferribacteres* phyla), as well as *Oscillospira* genus (belonging to the *Firmicutes* phylum) lowered in rats fed the HF diet. Finally, *Bacteroides uniformis* species increased, whereas *Bacteroides acidifaciens* species decreased in the HF group. The authors suggested that the HF diet prompted hepatic steatosis concomitant with the induction of microbiota dysbiosis.

As in the case of studies performed using very HF diets (60% of energy from fat), other studies have been addressed in rats. Mei et al. [24], fed rats either a normal chow diet (control group; 10% of calories from fat) or a HF diet (HF group; 45% energy from lard-based fat) for 150 days. The HF group showed moderate hepatic steatosis, as a disorder in globular structure was found and the cytoplasm was full of small lipid droplet vacuoles. Moreover, scattered chronic inflammatory cells were also observed. Regarding microbiota, *Firmicutes* increased in the HF group while *Bacteroidetes* diminished when compared to the control group, thus resulting in a significantly lower *Bacteroidetes/Firmicutes* ratio. The changes observed in the overall composition of gut microbiota induced by a HF diet, in comparison with a standard chow diet, may act as an important mediator in the etiology of NAFLD and related metabolic diseases by increasing inflammatory cytokines and causing lipid metabolism deregulation.

Feng et al. [34] fed Sprague–Dawley rats either a NF diet or a HF diet (45% of energy as fat from lard) for 16 weeks. At the end of the experimental period, the authors observed greater liver weight and hepatic lipid content after the HF diet feeding. Hepatic damage was detected as the ALT and AST concentrations were elevated, as well as metabolic endotoxemia revealed by the increased plasma LPS concentrations in the HF-fed rats. This fact contributed to a pro-inflammatory scenario, confirmed by higher serum TNF-α, intestinal (ileum) TLR4 protein expression or NF-κB activation. Furthermore, the number of CD8+ T lymphocytes in the lamina propria of the ileal mucosa was also augmented showing an immune response. Importantly, intestinal barrier integrity was also reduced in the rats offered the HF diet. Indeed, tight-junction protein expression levels, ZO-1 and occludin, were reduced in distal ileal tissues from HF diet-fed rats increasing intestinal permeability.

As observed in other papers included in this review [43,45,47], Feng et al. [34] reported that bacterial richness and alpha diversity were similar in both experimental groups, as suggested by Shannon index. As far as gut microbial composition is concerned, at phylum level HF diet led to an increase in *Actinobacteria*, *Proteobacteria*, and *Deferribacteres*, and to a decrease in *Spirochaetae*. Moreover, at genus level, increases in *Collinsella*, *Streptococcus*, *Gemella*, and *Elusimicrobium* genera and decreases in *Treponema* or *Quinella* genera were observed after HF diet feeding.

Recently, Duan et al. [45] have analyzed the effects of HF feeding by using male BALB/c mice. Animals were divided in two experimental groups: one fed a control diet and the other a HF diet (45% fat, mainly lard) for 10 weeks. The authors observed an increase in the weight and lipid droplet deposition in the animals fed the HF diet. Concerning microbiota, Shannon, Simpson, and Chao indices, all of which are used to analyze alpha diversity, were calculated. In contrast to other studies, no significant changes were observed. However, an increase in *Firmicutes*, (in which *Allobaculum spp*. was the dominant strain), accompanied by a decrease in *Bacteriodetes,* and thus a significantly lower *Bacteroidetes/Firmicutes* ratio, were observed in the HF group. Among *Bacteroidetes*, the main family levels were *Rikenellaceae*, *Prevotellaceae*, and *Bacteroidales S24_7*. Finally, an increase in *Pseudomonas* and *Lachnoclostridium* was also found.

Using KO mice for sirtuin 3 (SIRT3KO), Chen et al. [49] carried out a study feeding WT and SIRT3KO mice either a control diet or a HF diet (45% fat, mainly lard). At the end of the experimental period (18 weeks), higher levels of serum transaminases and two markers of inflammation (TNF-α and IL-6) were observed in animals of both HF diet-fed groups (WT and SIRT3KO). Regarding liver, histological analysis revealed an increase in steatosis grade, hepatocyte ballooning, lobular inflammation, and NAS between the control and the HF diet-fed groups. No changes were appreciated regarding alpha diversity (Chao1 and Shannon indices). By contrast, differences in beta diversity, which reported differences in bacterial clusters among groups, were revealed by PCoA. Based on these results, the authors reported that SIRT3 deficiency was associated with the alteration of the microbiota.

When fecal microbiota was analyzed, an increase in *Deferribacteres* phylum was observed in HF diet-fed SIRT3KO mice compared with HF diet-fed WT mice. Moreover, in mice fed the HF diet, increases in *Desulfovibrionaceae* family and *Desulfovibrio* genus were observed in the SIRT3KO animals. In addition, an increase in *Oscillibacter, Mucispirillum*, and *Parabacteroides* and a decrease in *Alloprevotella* were also appreciated in these same groups. Intestinal permeability was greater in HF diet-fed mice, and even higher in the SIRT3KO group. The mRNA expression of the gut permeability related CB1 receptor was also increased by HF feeding, this expression being higher in mice with SIRT3 deficiency than in WT. As far as CB2 receptor mRNA level is concerned, a decrease was observed in HF diet-fed SIRT3KO mice compared to mice fed the control diet.

Regarding inflammation, HF treatment increased the gene expression of *Tnf-α*, *Il-1β* and *Il-6* in the colon of both the SIRT3KO and WT mice when compared to the groups fed the control diet. This effect was greater in SIRT3KO mice. Furthermore, interleukin-10 (*Il-10*) mRNA expression was diminished in both HF groups. Based on these results, the authors concluded that SIRT3 play an essential role in maintaining gut permeability and inhibiting inflammation. Moreover, the authors demonstrated the role of SIRT3 in the amelioration of NAFLD via gut microbiota.

Other diets with a different amount of fat have also been used. Thus, Monteiro et al. [29] carried out a study in C57BL/6 male mice fed either a normolipidic diet (control), or a HF diet containing 38% lipids (11.4% *w*/*w* soybean oil and 88.6% *w*/*w* lard). At the end of the experimental period (9 weeks), the authors observed through histological analysis that the HF diet induced greater lipid accumulation in the liver.

Regarding the gut microbiota profile, *Firmicutes* phylum size was more than double than that of *Bacteroidetes*, whose size decreased in 63%. Moreover, the HF diet also increased *Verrucomicrobia* phylum (+55%) while reducing *Proteobacteria* (−65%). Similarly, the HF diet induced modifications in the cecal microbiota genera *Akkermansia, Helicobacter, Bacteroides, Oscillospira,* and *Butyricimona.* More specifically, the *Akkermansia* genus doubled its expansion in the gut, while *Helicobacter* and *Bacteroides* suffered a severe decrease. To know the effect of HF diet on endotoxinemia and lipid metabolism, biochemical parameters were assessed. Thus, greater LPS concentration was found in plasma from HF mice. High LPS levels are related to higher intestinal permeability, which triggers chronic inflammation with additional consequences, such as dyslipidemia or liver fat accumulation.

More recently, in the study reported by Li W et al. [40] male C57BL/6J mice were divided into two groups and fed either with a control diet or a HF diet (providing 30% of energy as lard) for 12 weeks. HF-fed mice displayed higher liver weight than control mice. As expected, HF feeding significantly increased the hepatic TG content. In addition, the HF diet induced significant increases in ALT and AST activities, which indicate substantial hepatocellular injury. Also, long-term consumption of HF diet caused damage-related changes, including vacuolation, degeneration, and the loss of cellular boundaries.

Moreover, an obvious difference in the composition of the gut microbiota between mice in the control and HF groups was observed. One of the most interesting changes was that mice fed the HF diet had less *Bacteroidetes* and more *Saccharibacteria, Proteobacteria*, and *Firmicutes* compared with the control group. At the class level, HF diet-fed mice showed fewer *Deltaproteobacteria* and more *Bacilli*. The abundance of microbes in the family level showed that this group had fewer *Porphyromonadaceae* and more *Lactobacillaceae, Helicobacteraceae, Coriobacteriacea*, and *Ruminococcaceae* compared with the control group.

## 3. Studies Carried Out by Using Other Lipid Sources

Apart from lard, other sources of dietary fat, such as milk, butter, cocoa butter or soybean have been used to induce liver steatosis in animal models (Table 2). In the study reported by Zhuang et al. [50] C57BL/6J mice from both sexes were fed either a low-fat diet (LF group, 10% of energy from fat) or a HF diet (45% of energy from milk-based fat) for 10 weeks. Then, the animals fed in the HF diet were maintained on the same diet for 15 additional weeks. As expected, significantly higher body weights were found in the HF groups of both sexes at the end of the experimental period when compared to the LF groups. As far as liver is concerned, a similar pattern was appreciated regarding the weight of this organ. When liver TG content was determined, this parameter was significantly increased in the HF-fed groups in both sexes. Regarding liver inflammatory status, higher mRNA levels of *Tnf-α* and *C-C motif ligand 2* (*Ccl2*) were found in males in the HF group when compared to the LF group. By contrast, in female mice, *Ccl2, Tnf-α* and *Il-6* gene expressions were greater in the HF group.

HF feeding induced a significant decrease in microbial richness when compared to that found under LF feeding conditions. In males, a reduction in the *Firmicutes/Bacteroidetes* ratio was observed, along with a lower relative abundance of *Proteobacteria*. In females, a similar pattern was also appreciated, although the reduction in *Proteobacteria* abundance was not as marked as in males. Moreover, an increase in *Verrucomicrobia* was also found in these animals. HF feeding also elevated circulating LPS levels, as well as diminished villus and crypt lengths in the cecum section.

Based on the results obtained, the authors concluded that a milk fat-based HF feeding induces intestinal microbiota dysbiosis, and that under these conditions, circulating LPS levels are enhanced, leading to an inflammatory status. Moreover, the authors highlight the potential influence of this gut microbiota dysbiosis derived inflammatory status on liver homeostasis impairment.

Also using milk-based fat, Natividad et al. [59] fed male C57BL/6J mice either a purified control diet or a HF diet (HF, 38% fat, mainly milk fat) for 9 weeks. The analysis of liver histology revealed that total hepatic TG were significantly elevated in the HF-fed group. Also, a significant increase in serum AST and ALT concentrations was observed after HF feeding.

When gut microbiota was analyzed, lower abundance of bacteria belonging to the genera *Ruminococcus, Bifidobacterium*, and *Parabacteroides* and to *Akkermansia muciniphila* species was appreciated in HF diet-fed mice. Moreover, higher abundance of bacteria from *Dorea* and *Sutterella* genera and *Ruminococcus gnavus* species was also observed in this group. Generally, these data showed that HF diet has a significant impact on microbiota composition.

In the study reported by Foster et al. [53], male Wistar rats had free access to either a control diet or to a HF diet rich in saturated fat (45% fat from cocoa butter), for 8 weeks. At the end of the experimental period, the authors found increased liver TG content and plasma ALT levels in the group fed the HF diet when compared with control animals. Similarly, negative effects of this feeding pattern regarding liver injury and inflammation were also observed. In this regard, HF diet induced increases in mRNA expression of gene markers of liver fibrosis such as α-smooth muscle actin (*Sma/Acta2*), transforming growth factor-β (*Tgfb1*), and collagen-α-1 (*Col1a1*). Moreover, greater gene expression on markers of inflammation such as Caspase-1 (*Casp1*), and endoplasmic reticulum stress such as spliced X-box binding protein-1 (*Xbp1s*), glucose-regulated protein *78* (*Grp78*), *C/EBP* homologous protein (*Chop*), growth arrest, and DNA damage inducible protein 34 (*Gadd34*) were also found in these animals. Regarding microbiota, the control group showed significantly greater amounts of *Bacteroidetes* and *Verrucomicrobia* and significantly lower amount of *Firmicutes* than rats fed the HF diet. There were also fewer *Lactobacillus* than in the control group.

Baldwin et al. [51] fed male C57BL/6J mice a NF diet (10% of energy from fat), or a HF diet (34% of energy from fat, mainly butter) for 11 weeks. Mice fed the HF diet showed greater liver weights, TG amounts and visual lipid staining, compared with the LF group. By contrast, *Tnf-α* was the only pro-inflammatory gene which was significantly increased in the ileal mucosa of HF diet-fed mice. To assess the intestinal barrier function, the localization of the proteins ZO-1 at the apical surface of the ileal epithelium was measured. When compared to the LF controls, impairment in this parameter was found in the groups fed the HF-sugar diets.

Then, the abundance of several mucosal sulfidogenic bacteria was measured. In this case, HF diet did not increase the abundance of any of the genes associated with sulfur metabolism in ileal or colonic mucosa compared to LF control. However, HF feeding induced decreases in *Firmicutes*, *Actinobacteria*, and *Tenericutes* phylum. These data suggest that several bacteria were significantly altered by HF diet feeding, causing a dysbiosis in microbiota.

In another study addressed by Collins et al. [52] using soybean as dietary lipid source, male C57BL/6J mice were fed either a normal-fat diet (NF; 10% of energy from fat) or a HF diet (44% of energy from fat) for 16 weeks. Although fat in HF group was mainly polyunsaturated, mice from this group showed greater liver weight and TG content compared to the NF group.

When the integrity of intestinal mucosa, which is frequently disrupted under these feeding conditions, was studied, impaired localization of ZO-1 in the apical area of the ileal epithelium surface was found in the HF group compared to the NF group. The activity of intestinal myeloperoxidase (MPO), but not alkaline phosphatase, was greater in the intestinal mucosa of the HF diet group compared to the NF diet group.

Regarding the inflammatory genes measured in ileum and proximal colon mucosa, cluster of differentiation 11c (*Cd11c*) and cluster of differentiation 68 (*Cd68*) increased by HF diet in the proximal colon. HF diet group also showed higher mRNA levels of *SCFA receptors* in the proximal colon mucosa compared to the NF group. Furthermore, HF feeding significantly reduced alpha diversity, compared to the NF diet. In this line, *Lachnospiraceae* family from the *Firmicutes* phylum decreased in the HF diet-fed group.

Zhou et al. [57] carried out a study in male C57BL/6J mice that were divided into five experimental groups: a control group fed a low-fat diet, a group fed a HF diet containing 20% (w/w) rapeseed oil, and three groups fed a diet containing medium length chain TG (MLCT) (10%, 20% or 30% w/w) for 6 weeks. The authors found a decrease in food consumption in the three groups treated with MLCT, but only the 20% MLCT group showed a significant difference in this parameter when compared with the HF group. Regarding hepatic TG content, only the animals on the 30% MLCT diet showed a significant reduction in this parameter.

Intake of the MLCT containing HF diet significantly lowered the proportion of *Firmicutes* to *Bacteroidetes* and decreased the relative abundance of *Proteobacteria,* which can be attributed to the weight loss observed. Mice fed the diets containing MLCT showed an increase in the total SCFA content in feces.

In another study, Milard et al. [60] aimed to know whether milk polar lipids (MPL) within a HF diet affected the gut microbiota. For this purpose, 8 week-old male C57Bl/6 mice were distributed into 4 experimental groups during 8 weeks: normal diet (ND), HF diet (HF) without MPL (21% palm oil + 1.4% of anhydrous milk fat), MPL1 group fed with a HF diet (21% palm oil + 0.7% of anhydrous milk fat + 1.9% of MPL-rich ingredient) and MPL2 fed with a HF diet (21% palm oil + 3.8% of MPL-rich ingredient). Thus, the HF diets had different quantities of MPL (0%, 1.1% or 1.6%), but the same quantity of milk TG (1.4%).

ND mice showed lower body weight gain than HF mice. This increase was totally prevented by MPL2. Liver weight remained unchanged, except for animals from MPL2 group, in which it was smaller than that of ND mice. When TG and polar lipids were measured in liver, no differences among groups were observed. HF and MPL2 groups showed lower hepatic macrophage infiltration than the other groups, as demonstrated measuring *F4/80* mRNA levels and by immunohistochemistry.

No effects on gut barrier function were detected. In the small intestine no differences in crypt depth, villus length, or mucus cell numbers were observed and in the colon, MPL2 animals showed higher crypt depth when compared to HF and MPL1. In these groups, the total amount of bacteria and the *Firmicutes/Bacteroidetes* ratio were not modified by any treatment. The only change induced by the HF diet was the increase in *Bifidobacterium animalis*. In pooled data from both MPL groups, *Akkermansia muciniphila* was more abundant than in the HF group. When MPL1 group was compared to HF group, *Bifidobacterium animalis* increased. In the case of MPL2, a reduction in *Lactobacillus reuteri* was observed. The authors concluded that it was not possible to determine a direct impact of gut microbiota on anthropometric and metabolic parameters because no correlations were observed.

Finally, Tian et al. [30] addressed a study carried out in male Sprague–Dawley rats. They distributed the animals in four groups: one group fed with a NF diet, providing 10% of energy from fat (control group), and the others groups fed with a Western style lard-rich diets providing 45% of energy from fat and 2% cholesterol, supplemented with 10% fish oil (FO) or 5.5% perilla oil (PO) or without supplementation (HF), for 16 weeks. HF feeding induced a significant increase in liver weight and ALT values. Both oils partially prevented the increase of ALT values. Moreover, HF feeding induced significant hepatic inflammation, as shown by the increase in hepatic mRNA expression of *Tnf-α*, *Il-1ß, Il-6,* and *Tlr4*. These increments in mRNA expression were partially prevented by both oils in the of *Il-1ß* and totally prevented for *Tnf-α*, *Il-6,* and *Tlr4.*

The study also showed that the composition of the gut microbiota was significantly altered by HF diet consumption. In this regard, an increase in *Bacteroidetes* and a decrease in *Firmicutes* were observed in all animals fed a high-fat diet, thus resulting in a significantly higher *Bacteroidetes/Firmicutes* ratio. Regarding the phylum *Firmicutes*, the three HF diets induced a lower abundance of *Achnospiraceae* and *Ruminococcaceae* and a higher abundance of *Peptostreptococcaceae*. In the phylum *Bacteoidetes*, the three HF diets increased *Prevotellaceae*, *Bacteoidaceae*, and *S24-7.* FO and PO groups showed lower *Prevotellaceaes* than HF group. As far as *Firmicutes* genus was concerned, animals fed HF diet showed lower *Ruminococcus, Oscillospira*, and *Clostridium*. Although the population of *Lachnospiraceae* was much lower in animals fed the HF diet than in the control group, the relative populations of *Roseburia* genus were higher in this group. Also, HF group had more *Blautia* than the other groups. Similarly, in the *Ruminococcaceae* family, HF diet augmented the population of *Faecalibacterium*, compared with the other groups; in the *Lactobacillaceae* family, the population of the only abundant genus *Lactobacillus* was higher in HF group than in the other groups. Moreover, HF feeding raised the relative abundance of genus *Escherichia* and *Sutterella* in phylum *Proteobacteria* and genus *Bifidobacterium* in phylum *Actinobacteria*, when compared with the other groups. Finally, FO group increase the abundance of *Akkermansia*.

The authors concluded that an unhealthy high-caloric diet can induce intestinal microbiota alteration, leading to systemic inflammation, which is thought to play a major role in the development of NAFLD. These alterations can partially be prevented by oils rich in polyunsaturated fat.

## 4. Studies with No Identified Dietary Lipid Source

Finally, there are other studies in which the type of dietary fat is not specified. This is the case of the study reported by Yamada et al. [56] who fed specific pathogen-free (SPF) C57BL/6J mice a standard diet until 8 weeks of age. During this experimental period, the gut microbiota of the animals was normalized. Then, the animals were fed a standard diet (C group) or a HF diet for 9 weeks. After this period, the animals fed in the HF diet were divided in two groups: one group received the diet for 9 weeks (HF group), while the other group was treated with an antibiotic cocktail from week 7 to week 17 in order to induce microbiota depletion (HF + Abx group). The fat content of the standard diet was 12% of the energy (cereal germ and soybean), while in the case of the HF diet, this was 72% of the energy (with high content of saturated fatty acids and cholesterol).

Histological analysis showed greater fat droplet accumulation, ballooning, fibrotic extensions, and inflammatory cells in the livers of the HF group when compared to the other groups. Also, hepatic TG content was significantly greater in the HF group in comparison with the C and HF + Abx groups. Moreover, serum ALT and AST levels were also higher in this group. The authors also found a sharp increase in the gene expression of several inflammatory and fibrosis markers, such as *Tnf-α*, *Il-1β*, *α-Sma* and α1 type 1 collagen (*Col 1α1*) in the HF group when compared to the C and HF + Abx groups

When gut microbiota composition was analyzed, while significantly reduced gut microbiome amount was found in the feces of the animals in the HF + Abx group, no differences in this parameter were found between the C and the HF groups. As far as the HF group is concerned, the dysbiosis induced by this feeding pattern included lowered *Bifidobacterium, Enterococcus*, and *Bacteroides* abundance and increased *Clostridium* XIVa and XIII subclusters. In the case of the HF + Abx group, the antibiotic treatment led to a dominating presence of *Enterococcus* genre in the gut microbiota. With regard to the effects of HF feeding-induced dysbiosis on the gut microbiota metabolic activity, gut metabolome analysis revealed that long-chain fatty acid (>14 C) and ω-6 unsaturated fatty acid pathways were constantly elevated in HF group compared with the C group. Then, to determine if the accumulation of saturated long-chain fatty acids and ω-6 unsaturated fatty acids was related to liver inflammation, the amount of F4/80^high^CD11b^high^ migratory macrophages was measured in this organ, as well as the plasma levels of MCP-1. In this regard, significant increases in both parameters were found in the HF group.

Based on the results obtained, the authors concluded that the changes in gut microbiota were involved in the development of HF diet-induced NASH. Moreover, the authors suggested that the changed luminal metabolic profile, along with the activation of migratory macrophages and their subsequent migration to the liver, could underlie the HF diet induced liver inflammation.

Xu et al. [55] carried out a study with ten-week-old male C57BL/6J. Animals were fed normal chow diet (NC) and two HF (containing 60% fat by energy) for ten weeks. Increased TG, NEFA and total cholesterol levels were appreciated in serum of C57BL/6J mice fed a HF diet. These changes were accompanied with an increase in liver TG, NEFA content, and lipid infiltration determined by Oil Red O-staining. Moreover, gene expression of liver fatty acid transporter protein (*Fabp*), *Cd36*, *Srebp1c,* and *Fasn* were up-regulated. Inflammatory parameters were also analyzed in this study and, as expected, serum TNF-α was elevated in HF animals. In the case of mRNA expression *Tnf-α, Il-1β* and *Cox-2* were also higher.

Regarding the small intestine, a marked decrease in length and a different morphology (the villus length in the proximal jejunum was reduced) were observed in HF group. Moreover, a down-regulation of mRNA expression of occludin was also reported. PCoA revealed a different cluster of microbiota between both groups. In phylum level, a decrease in *Cyanobacteria* accompanied with an increase in *Tenericutes* and *Actinobacteria* was observed.

Ishioka et al. [54] reported a study where they analyzed the effect of different experimental diets in the induction of steatohepatitis. For this purpose, male and female C57BL/6 mice were fed normal chow diet (control group) or a high-fat diet (HF; 60% calories from fat) for 8 weeks. Histologically, the authors observed hepatic steatosis in the HF diet-fed mice. However, the HF diet induced steatosis without inflammation (simple steatosis). Concerning hepatic lipid levels, TG, total cholesterol, and free fatty acids level were augmented in the liver of mice fed in HF diet. When analyzing the intestinal microbiota, the authors observed that *Firmicutes/Bacteroidetes* ratio was significantly increased in the obese HF group. They studied the phylum *Firmicutes* in more depth, because it includes at order level *Clostridiales* and *Lactobacillales*, which produce SCFAs and lactate, respectively. Thus, HF diet increased the abundance of lactate-producing bacteria (*Lactobacillales*). Regarding the *Bacteroidales* order, *Bacteroides* and *Parabacteroides* were major components at genus level. At species level, *Parabacteroides goldsteinii*, which is associated with anti-inflammation in high-fat-diet-induced obesity, was diminished. Concerning the diet-induced inflammation, Ishioka et al. analyzed IL-17 expression, which is a gut microbiota-mediated cytokine. The results revealed that in mice receiving control or HF diets, IL-17 cytokine was not detected in cells from the small intestine. Consequently, the authors did not observe that the gut microbiota and the immune response varied considerably between both groups.

## 5. Mechanisms of Action

The main limitation of most of the studies described in this review is that unfortunately the authors show the changes induced by HF feeding on liver and gut microbiota, without proposing any explanation to establish a relationship between these two types of changes. Nevertheless, several authors have proposed some mechanisms of action underlying the effects on liver steatosis of changes in gut microbiota induced by HF feeding. Among them, dysbiosis-induced deregulation of the gut barrier function, increased hepatic and intestinal inflammation, and elevated metabolites (lipopolysaccharides, short-chain fatty acids, bile acids and ethanol) produced by the microbiota seem to be the most important ones [61,62].

### 5.1. Mechanisms Related to Gut Permeability

Increased gut permeability promotes bacterial translocation and the passage of detrimental bacterial products to the circulation, which then reach the liver [63,64]. This fact is related to hepatic inflammation, which takes place in NAFLD, essentially mediated by nucleotide-binding and oligomerization domain (NOD)-like receptors (NLRs) and TLRs [62].

### 5.2. Intestinal Architecture Modification

The intestinal epithelium allows molecules to pass through by non-mediated diffusion. The solute absorption across the intestinal epithelium can be transcellular, through the enterocyte, crossing the apical and basolateral membranes, or paracellular, crossing the epithelium between cells. In fact, endotoxins, as well as other bacterial products, pass through the intestinal mucosa via a paracellular pathway [65]. Epithelial tight junctions are located at the apical part of the lateral membranes of epithelial cells, creating a dynamic structure, which opens or closes the paracellular route in response to several stimuli [66]. In this scenario, three groups of molecules that belong to tight junctions are responsible for gut permeability: occludin, claudins, and ZO-1. It is known that claudins form tight-junction pores for smaller molecules, whereas occludins and ZO-1 play a role in enhancing tight junctions permeability to macromolecules [67]. Regulation of the paracellular flux is linked to the actomyosin cytoskeletal restructuring triggered by pro-inflammatory cytokines, such as TNF-α and IL-1β [68]. Moreover, E-cadherin and β-catenin are also essential for the organization of the tight-junction complex, and for subsequent maintenance of intestinal architecture.

NAFLD has been associated with increased gut permeability, and with small intestinal bacterial overgrowth (SIBO) [13]. In addition, the progressive disruption of the tight junctions could explain the contribution of the intestinal architecture to NAFLD progression.

Among the studies previously described in this review, Chen et al. [44] and Baldwin et al. [51] observed mucosal damage in animals fed HF diets. Other authors, who studied this mechanism in more depth, detected a significant decrease in the tight junctions [23,35,50]. Zhuang et al. [50] noticed a significant decrease in villus and crypt lengths in the cecum section. Indeed, the determination of the intestinal villus to crypt ratio is a common histomorphometric method used to assess the intestinal epithelium state.

### 5.3. Inflammation

**Figure 1:**
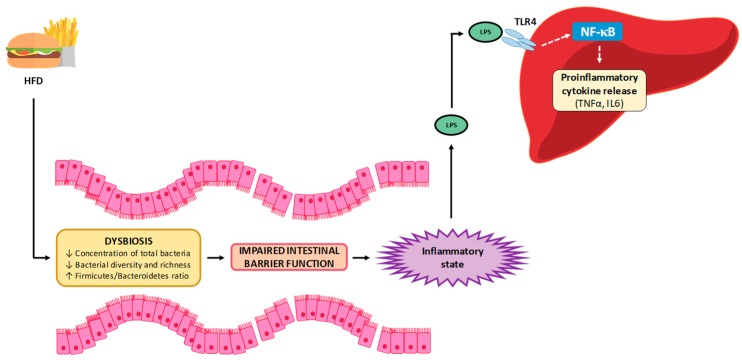
Involvement of inflammation, induced by changes in microbiota associated with high-fat feeding, in liver steatosis development. (Based on Porras et al., 2018 [70]). HFD: High-fat diet, LPS: lipopolysaccharide, TLR-4: Toll-like receptor 4, NF-κB: Nuclear factor κB, TNF-α: Tumor necrosis factor α, IL-6: Interleukin-6.

A low-grade inflammatory state has been described in NAFLD, which has been proposed to be related to increased intestinal permeability and a greater delivery of microbial products to circulation [69]. One such product is LPS, which once in the bloodstream can reach the liver, binding to TLR4 and activating an inflammatory cascade in which NF-κB is the last effector [62,70]. This activation results in a greater production of inflammatory cytokines, such as TNFα and IL-6, inducing an inflammatory state in the liver, which is a well-known contributor to the development and/or progression of NAFLD [71] (Figure 1). Moreover, intestinally derived LPS is also involved in the development of liver fibrosis. It has been shown that activated hepatic stellate cells, which represent the source for collagen production during liver fibrosis, express TLR4 and thus are activated by LPS [72].

Among the studies analyzed in this review, increased plasma LPS levels were widely reported in those in which diets with a lard-based fat content, ranging from 60 to 38% of energy, were used [23,26,28,29,35]. Moreover, this effect was also observed in a study carried out by using 45% of milk-based fat [71]. In addition, increased hepatic *Tnf*-α mRNA expression was also observed in a study carried out using a diet with a lard-based fat content of 60% [26]. Finally, in a study in which a diet with a fat content of 60% from unknown origin was used, elevated TNF-α and IL-6, as well as activation of NF-κB, were also reported [58].

Furthermore, Collins et al. [52] observed an increase in the intestinal myeloperoxidase activity of neutrophils, a biochemical parameter that allows one to quantify inflammation in the gut [73]. Similarly, Chen et al. [44] detected intestinal inflammation in HF treated animals through increased HMGB1 levels in the gut and exosomes, a pro-inflammatory cytokine secreted by immune cells.

### 5.4. Short-Chain Fatty Acids and Ethanol

Gut microbiota modulates host biology in numerous ways, influencing on the maintenance of host health. However, several known microbial metabolites have not yet been functionally characterized, so the mechanisms underlying these highly mutualistic interactions are still poorly understood [74].

Diet-dependent microbial products can be directly generated by digestion or fermentation of dietary components, or generated from products of host metabolism biochemically modified by the gut microbiota. SCFAs are volatile fatty acids produced through the bacterial fermentation of undigested polysaccharides [75]. Members of *Firmicutes* are the main producers of butyrate, whereas *Bacteroidetes* produces mostly acetates and propionates. Acetate is the most abundant SCFA in the colon, and it is a byproduct of undigested polysaccharide fermentation by most enteric bacteria. Also, several acetogenic bacteria can synthesize acetate via the Wood–Ljungdahl pathway [76]. Furthermore, most propionate is formed by *Bacteroidetes* while using succinate as substrate.

SCFAs are the main energy source for colonic epithelial cells, thus contributing to maintaining intestinal integrity [62]. However, a significant amount of these SCFAs are also detected in the portal vein, reaching the liver; this being the main site for the clearance [77]. When using mice with nonalcoholic steatohepatitis, it was shown that restoration of butyrate in this organ elevates the expression of insulin receptor substrate 1 to improve hepatic insulin sensitivity and steatosis [78]. Moreover, propionate can reduce hepatic de novo lipogenesis. By contrast, acetate can be used as a lipogenic substrate [79], suggesting that the specific pattern of SCFAs may be crucial for understanding their implication in NAFLD [62].

In this regard, few manuscripts have explored SCFAs production by microbiota after HF diet feeding reporting contradictory results. Thus, by using C57BL/6J mice fed a diet supplying 60% of energy as fat, Porras et al. [35] found that SCFAs production in HF-fed mice was lower than in control mice. These results correlated positively with the lower expression of intestinal tight-junction proteins claudin 1 and occludin. Nevertheless, Li et al. [40] found no changes in SCFAs production after HF diet (30% of energy as fat) feeding. Therefore, the amount of dietary fat might be important in this effect. Moreover, Collins et al. [52] found increased gene expression of SCFA receptors in mice fed a high soybean oil diet.

Several bacteria, such as Escherichia coli, produce alcohols that can derive in ethanol, thus inducing hepatic damage in a similar way to alcoholic fatty liver disease [35]. Thus, Porras et al. [35] observed this effect in mice fed a diet which provided a high amount of fat from lard.

### 5.5. Bile Acid Composition

Primary bile acids found in biliary secretion are metabolized by microbiota and transformed into secondary bile acids, which are more hydrophobic and consequently more cytotoxic [80]. They can be reabsorbed by means of enterohepatic circulation, thus reaching the liver. In this organ they induce negative effects such as mitochondrial dysfunction, endoplasmic reticulum stress and inflammation [81], which are described as metabolic features of liver NAFLD. Microbiota composition has a clear influence on bile acid synthesis [82]. Thus, bile acids are directly deconjugated by *Bacteroides*, *Clostridium*, and *Eubacterium*, which show bile salt hydrolase activity [83]. Moreover, bacteria can affect apical sodium-dependent bile acid transporters, thus preventing active reuptake of bile acids [84].

The studies reviewed here include one reported by Tang et al. [46] which showed increased taurocholic acid but reduced taurohyodeoxycholic acid and ursodeoxycholic acid in the liver from rats showing NAFLD induced by a HF diet. In addition, the deconjugated form of cholic acid increased, while the deconjugated forms of α-hyodeoxycholic acid and ω-muricholic acid were diminished in the gut. Finally, serum concentration of taurocholic acid increased, while those of α-hyodeoxycholic acid and taurohyodeoxycholic acid decreased. When correlations were carried out between changes in microbiota and changes in bile acids, the authors observed that *Bacteroidetes* was positively correlated with α-hyodeoxycholic acid, *Prevotella* was negatively correlated with cholic acid and *Parasutterella* was positively correlated with ω-muricholic. According to these results, it can be stated that the altered bile acid profile is highly correlated with gut dysbiosis.

### 5.6. Other Mechanisms

**Figure 2:**
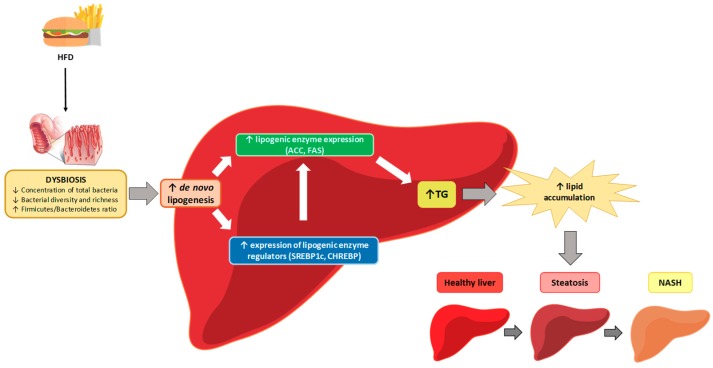
Effects of high-fat diet derived dysbiosis in hepatic in hepatic de novo lipogenesis and liver lipid accumulation (Based on Parnell et al. [70]. HFD: High-fat diet, ACC: acetyl-CoA carboxylase, FAS: fatty acid synthase, SREBP1c: sterol regulatory element-binding protein 1c, CHREBP: carbohydrate responsive element-binding protein, NASH: nonalcoholic steatohepatitis.

Some authors have reported that dysbiosis can modify hepatic TG metabolism, by increasing the activity of acetyl-CoA carboxylase (ACC) and fatty acid synthase (FAS), which are the main enzymes of de novo lipogenesis (Figure 2) [70,85]. In addition, several types of bacteria can also increase this metabolic pathway by reducing the production of fasting-induced adipocyte factor (FIAF), a protein with inhibitory effect through the transcriptional factors SREBP-1c and ChREBP [86]. The increase in de novo lipogenesis leads to enhanced fatty acid availability for hepatic TG synthesis and thus to the hepatic accumulation of this lipid species. Among the studies described in the present review, Xu et al. [55] found higher *Fasn* expression in mice fed a HF diet providing 60% of energy from fat.

## 6. Concluding Remarks

An overview of the present review shows that liver steatosis can be induced not only by lard, but also by other saturated dietary lipid sources, or even by soybean oil. By contrast, omega-3 fatty acids have been shown to prevent livers steatosis induced by saturated fat. Most of the studies have found a reduction in microbiota diversity, independently of the percentage of energy provided by fat. When lard is the main source of dietary fat, the most important changes in microbiota composition is an increase in *Firmicutes* [24,25,30,33,34,38,40,41,46,47], as well as in the *Firmicutes/Bacteroidetes* ratio [22,24,25,26,27,29,30,36,40,41,45,46,47,48], although a decrease in this phylum has also been observed by other authors [32,39]. Interestingly, this is not the profile observed when other dietary fat sources are used. Thus, the *Firmicutes/Bacteroidetes* ratio shows a decrease when feeding animals with rapeseed oil [51], cocoa butter [54] and milk-based fat [58], and *Firmicutes* richness decreases when butter is used as the main dietary fat source [52]. This fact raises the question of the involvement of changes in this ratio in the induction of liver steatosis by HF feeding.

Other phyla have also been modified by high-fat diets, but a consensus is not reached. Thus, whereas in some studies increases in *Verrumicrobia* [29,36,38,53] or *Tenericutes* [30] have been described, in others decreases have been found [33,39,51]. The same situation takes place at the genus level. Whereas increases in *Bacteroides* [25,30,47] or Prevotella [25,30] have been reported by some authors, decreases have been described by others [27,28,29,45,56]. Finally, *Akkermansia muciniphila,* a bacteria species related to obesity and type 2 diabetes, is also affected by dietary fat, increasing [29,30] or decreasing [23,39] depending on the studies. The differences observed among the reported studies can be due to differences in experimental design. Nevertheless, in the discussion sections of these studies no explanations concerning this issue are provided.

A question that can arise is whether changes observed in gut microbiota in these studies are just related to liver steatosis or they also associated with obesity. Due to the fact that in most of the reported studies, animals following a HF dietary pattern showed significantly greater body weight and/or body fat, it is difficult to give an answer to this question. Nevertheless, several changes in microbiota composition observed in studies where changes in body weight and body fat were not observed, were also found in other studies were these parameters increased.

It is important to emphasize that an important limitation of the reported studies is that the diets used show a lipid dietary composition that does not reflect that of human diets, even when the dietary pattern is unbalanced. Consequently, the extrapolation of the results obtained in the reported studies to humans is limited.

Regarding the potential mechanisms underlying the relationship between livers steatosis and changes in gut microbiota, it is important to point out that a great number of the reported studies do not address this issue. In other studies some mechanisms have been proposed: a) inflammation due to LPS [23,26,28,29,34,35,41], production of inflammatory molecules, such as TNF-α and IL-6 [19,26,58], mucosal damage [44,51], decrease in the tight junctions [23,35,50], decrease in villus and crypt lengths [50,55], acetate, butyrate and propionate production [35,57] and changes in bile acids [46].

## Figures and Tables

**Table 1:** Studies conducted in rodent models using lard-based high-fat diets.

Reference	Animal Model	% Fat	Main Fat Source	Changes in Gut Microbiota	Potential Mechanisms Involved in the Relationship between Changes in Microbiota and Liver Steatosis
Gauffin Cano et al. (2012) [19]	Male C57BL-6 mice (6–8-week-old)	60%	Lard	↓ *Lactobacillus, Bifidobacterium, Clostridium coccoides* and *Clostridium leptum* ↑ *Enterobacteriaceae*	↑ Production of TNF-α by macrophages and dendritic cells (in vitro, stool-induced)
Mei et al. (2015) [24]	Male Sprague–Dawley rats	45%	Lard	↑ *Firmicutes* ↓ *Bacteroidetes*	Not detailed
Seo et al. (2015) [25]	Male C57BL/6 mice (6-week-old)	45%	Lard	↑ *Firmicutes*/*Bacteroidetes* ratio ↑ *Bacteroides*/*Prevotella* ratio	Not detailed
Wang et al. (2015) [26]	Male C57BL/6J mice (10-week-old)	60%	Lard	↑ *Firmicutes/Bacteroidetes* ratio ↑ *Proteobacteria* ↓ *Actinobacteria*	↑ Hepatic *Tnf-α* mRNA induced by ↑ LPS levels in plasma
Leal-Díaz et al. (2016) [27]	Male C57BL/6 mice (5-week-old)	45%	Lard	↓ Bacteria diversity ↓ *Prevotella*, *Mucispirillum* and *Oscillospira*	Not detailed
Liu et al. (2016) [28]	Male Sprague–Dawley rats (5-week-old)	60%	Lard	↓ *Bacteroidetes/Firmicutes* ratio ↑ *Roseburia* and *Oscillospira* ↓ *Bacteroides* and *Parabacterioides*	↑ LPS levels in plasma
Monteiro et al. (2016) [29]	Male C57BL/6 mice	38%	Lard	↑ *Firmicutes, Verrucomicrobia* and *Akkermansia* ↓ *Bacteroidetes, Proteobacteria, Helicobacter* and *Bacteroides*	↑ LPS
Tian et al. (2016) [30]	Male Sprague–Dawley rats (8–9-week-old)	45%	Lard	↑ *Prevotella* and *Bacteroides* ↑ *Blautia*, *Faecalibacterium*, *Lactobacillus*, *Escherichia* and *Sutterella* ↓ *Ruminococcus*, *Oscillospira* and *Clostridium*	Not detailed
Tung et al. (2016) [31]	Male C57BL/6 mice (5-week-old)	45%	Lard	↓ *Firmicutes* ↑ *Bacteroidetes* ↓ *Clostridiales* ↑ *Bacteroidales* and *Sphingobacteriales*	Not detailed
Chiu et al. (2017) [32]	Male GF C57BL/6JNarl mice (3–4-week-old)	60%	Lard	↑ *Firmicutes*	Not detailed
Duparc et al. (2017) [33]	C57BL/6 WT mice (Hepatocyte-specific Myd88 KO mice)	60%	Lard	↑ Bacterial diversity ↑ *Firmicutes* ↓ *Tenericutes* ↓ *Sutterella* and *Allobaculum* ↑ *Ruminococcus* and *Oscillospira*	Not detailed
Feng et al. (2017) [34]	Male Sprague–Dawley rats (4-week-old)	45%	Lard	↑ *Actinobacteria*, *Proteobacteria* and *Deferribacteres* ↓ *Spirochaetae* ↑ *Collinsella, Streptococcus, Gemella* and *Elusimicrobium* ↓ *Treponema* and *Quinella*	↑ LPS levels in plasma ↓ Intestinal tight-junction
Porras et al. (2017) [35]	Male C57BL/6J mice (7-week-old)	60%	Lard	↓ Concentration of total bacteria ↑ *Helicobacter* expansion ↑ *Firmicutes/Bacteroidetes* ratio ↑ *Proteobacteria* ↑ *Clostridia*, *Bacilli* and *Deltaproteobacteria* ↓ *Bacteroidia*, *Erysipelotrichi* and *Betaproteobacteria*	↓ Intestinal tight-junction ↓ Acetate, butyrate and propionate SCFAs ↑ LPS and ethanol levels in plasma
Su et al. (2017) [23]	Male BALB/c mice (4–6-week-old)	60%	Lard	↑ *Firmicutes* ↓ *Bacteroidetes* ↑ *Helicobacter hepaticus* ↓ *Akkermansia muciniphila*	↑ LPS levels in plasma ↓ Intestinal tight junctions
Wang et al. (2017) [21]	Male ICR mice (4-week-old)	60%	Lard	↓ *Bacteroidetes/Firmicutes* ratio ↓ *Deferribacteres*	Not detailed
Xu et al. (2017) [22]	Male C57BL/6J mice (10-week-old)	60%	Lard	↑ *Firmicutes/Bacteroidetes* ratio ↑*Helicobacter marmotae*, *Odoribacter* and *Anaerotruncus*	Not detailed
Chen et al. (2018) [36]	Male Sprague–Dawley rats (6-week-old)	60%	Lard	↑ *Proteobacteria* and *Verrucomicrobia* ↓ *Bacterioidetes* and *Tenericutes* relative abundances	Not detailed
Jia et al. (2018) [37]	C57BL/6 J mice (6-week-old)	60%	Lard	↑ *Firmicutes, Eubacterium, Blautia*, *Clostridium, Lactobacillus* and *Escherichia* ↓ *Parasutterella*	Not detailed
Jing et al. (2018) [38]	Male C57BL/6J mice	60%	Lard	↓ *Firmicutes* ↑ *Verrucomicrobia*	Not detailed
Li et al. (2018) [39]	Male C57BL/6J mice (8-week-old)	60%	Lard	↑ *Firmicutes, Proteobacteria, Lachnoclostridium, Acetatifactor*, *Lactococcus, Romboutsia, Enterorhabdus* and *Dorea* ↓ *Actinobacteria, Bacteroidetes, Verrucomicrobia, Akkermansia, Olsenella, Barnesiella* and *Alloprevotella*	Not detailed
Li et al. (2018) [40]	Male C57BL/6J mice	30%	Lard	↑ *Candidatus, Saccharibacteria, Proteobacteria, Firmicutes, Bacilli, Lactobacillaceae, Helicobacteraceae, Coriobacteriacea* and *Ruminococcaceae* ↓*Bacteroidetes, Deltaproteobacteria* and *Porphyromonadaceae*	Not detailed
Martins et al. (2018) [41]	Female C57BL/6 mice (deficient in IL-23)	60%	Lard	↑ *Lactobacillaeceae and S24*-7 ↓ *Lachnospiraceae*	↑LPS levels in plasma
Wang et al. (2018) [42]	C57BL/6 J mice	60%	Lard	↑ *Clostridium leptum* ↓ *Bacteriodetes fragilis*	Not detailed
Wu et al. (2018) [43]	C57BL/6 J mice	60%	Lard	↑*Deltaproteobacteria, Deferribacteres*, *Desulfovibrionaceae*, *Deferribacteraceae*, *Porphyromonadaceae*	Not detailed
Chen et al. (2019) [44]	C57BL/6J WT mice	60%	Lard	↑ *Firmicutes/Bacteroidetes* ratio	↑Mucosal damage ↑ Co-localization of HMGB1 and CD63, in gut ↑HMGB1 in serous exosomes
	C57BL/6J ASC-KO mice	60%	Lard	↓ Bacterial diversity ↑ *Firmicutes/Bacteroidetes* ratio ↑ *Streptococcaceae*	↑Mucosal damage ↑ Co-localization of HMGB1 and CD63, in gut ↑ HMGB1 in serous exosomes
Duan et al. (2019) [45]	BALB/c mice	45%	Lard	↑ *Firmicutes, Allobaculum* spp, *Pseudomonas* and *Lachnoclostridium*. ↓ *Bacteriodetes, Rikenellaceae*, *Prevotellaceae, Bacteroidales (S24-7)*	Not detailed
Tang et al. (2019) [46]	Male Sprague–Dawley rats (6-week-old)	60%	Lard	↑ *Firmicutes* ↓ *Bacteriodetes*	Changes in bile acids (↑ taurocholic and ↓ taurohyodeoxycholic and urodeoxycholic acid contents)
Wu et al. (2019) [47]	Male C57BL/c mice	60%	Lard	↓ *Bacteroidetes/Firmicutes* ratio ↑ *Anaerotruncus*, *Streptococcus* and *Bacteroides*	Not detailed

ASC-KO: apoptosis-associated speck-like protein containing a C-terminal caspase recruitment domain knock-down, CD63: cluster of differentiation 63, GF: germ free, HF: high-fat, HMGB1: high mobility group box 1, ICR: institute of Cancer Research, IL-23: interleukin 23, LPS: lipopolysaccharide, SCFA: short-chain fatty acids, SPF: specific pathogen-free, TNFα: tumor necrosis factor α, WT: wild-type. ↑: significant increase, ↓: significant decrease.

**Table 2:** Studies conducted in rodent models using high-fat diets with fat of origins other than lard (butter, milk, plant-based, or unknown origin).

Reference	Animal Model	% Fat	Main Fat Source	Changes in Gut Microbiota	Potential Mechanisms Involved in the Relationship between Changes in Microbiota and Liver Steatosis
Baldwin et al. (2016) [51]	Male C57BL/6J mice (4-week-old)	34%	Butter	↓ *Firmicutes*, *Actinobacteria* and *Tenericutes*	↑ Mucosal damage
Collins et al. (2016) [52]	Male C57BL/6J mice	44%	Soybean oil	↓ *Lachnospiraceae*	↓ Intestinal mucosa integrity ↑ Cd11c, Cd68 and SCFA receptors mRNA levels
Foster et al. (2016) [53]	Male Wistar rats	45%	Cocoa butter	↑ *Bacteroidetes* and *Verrucomicrobia* ↓ *Firmicutes* and *Lactobacillus*	Not detailed
Tian et al. (2016) [30]	Male Sprague–Dawley rats (8–9-week-old)	45%	Lard (1/2) + FOH (1/2)	↑ *Prevotella* relative abundance ↑ *Akkermansia* ↓ *Ruminococcus*, *Oscillospira* and *Clostridium*	Not detailed
			Lard (3/4) + POH (1/4)	↑ *Prevotella* relative abundande ↓ *Ruminococcus*, *Oscillospira* and *Clostridium*	Not detailed
Ishioka et al. (2017) [54]	C57BL/6 mice	60%	Not specified	↑ *Firmicutes/ Bacteroidetes* ratio ↑ *Lactobacillales* (lactate-producing bacteria)	Not detailed
Xu et al. (2017) [55]	Male C57BL/6J (10-week-old)	60%	Not specified	↓ *Cyanobacteria* ↑ *Tenericutes* and *Actinobacteria*	Intestinal morphology changes ↓ Villus length (proximal jejunum) ↓ Occludin mRNA expression
Yamada et al. (2017) [56]	SPF C57BL/6J mice	72%	Not specified (high content of SFA and cholesterol)	↓ *Bifidobacterium, Enterococcus* and *Bacteroides* ↑ *Clostridium* (XIVa and XIII subclusters)	Not detailed
Zhou et al. (2017) [57]	Male C57BL/6J mice	20%	Rapeseed oil	↓ *Firmicutes* to *Bacteroidetes* proportion ↓ *Proteobacteria*	↑ Total SCFA content (feces)
Zhuang et al. (2017) [50]	C57BL/6J mice (4-week-old)	45%	Milk-based fat	↓ Microbial richness ↓ *Firmicutes*/*Bacteroidetes* ratio and *Proteobacteria* ↓ *Firmicutes*/*Bacteroidetes* ratio (females)	↓ Villus and crypt lengths (cecum section) ↑ LPS levels in plasma
Li et al. (2018) [58]	Male mice C57BL/6J(4-week-old)	60%	Not specified	↓ *Bacteroidetes* ↑ *Firmicutes*	↑ NF-κB activation and ↑ IL-6 and TNF-α production
Natividad et al. (2018) [59]	Male C57BL/6J	38%	Milk fat	↓ *Ruminococcus, Bifidobacterium, Parabacteroides* and *Akkermansia* ↑ *Dorea* and *Ruminococcus* gnavus ↑ *Sutterella* genera	Not detailed
Milard et al. (2019) [60]	Male C57Bl/6 mice	46%	Palm oil	↑ *Bifidobacterium animalis*	Not detailed
			Palm oil + 1.1% MPL	↑ *Bifidobacterium animalis* (vs. HF)	Not detailed
			Palm oil + 1.6% MPL	↓ *Lactobacillus reuteri* ↑ *Bifidobacterium animalis* (vs. C)	↑ Crypt depth (vs. HF)

Cd11c: cluster of differentiation 11c, Cd68: cluster of differentiation 68, HF: high-fat, FOH: fish-oil-rich diet, IL-6: interleukin-6, LCFA: long-chain fatty acid, MPL: milk polar lipids, LPS: lipopolysaccharide, NF-κB: nuclear factor kappa B, POH: perilla-oil-rich diet, SCFA: short-chain fatty acid, SFA: saturated fatty acid, SPF: specific pathogen-free, TNFα: tumor necrosis factor α, UFA: unsaturated fatty acid. ↑: significant increase, ↓: significant decrease.
